# Magnetic Resonance-Guided Stereotactic Body Radiation Therapy/Hypofractionated Radiation therapy for Metastatic and Primary Central and Ultracentral Lung Lesions

**DOI:** 10.1016/j.jtocrr.2023.100488

**Published:** 2023-02-25

**Authors:** Maria L. Sandoval, Austin J. Sim, John M. Bryant, Menal Bhandari, Evan J. Wuthrick, Bradford A. Perez, Thomas J. Dilling, Gage Redler, Jacqueline Andreozzi, Louis Nardella, Vladimir Feygelman, Kujtim Latifi, Stephen A. Rosenberg

**Affiliations:** a Department of Radiation Oncology, H. Lee Moffitt Cancer Center and Research Institute, Tampa, Florida; b Department of Radiation Oncology, James Cancer Hospital, The Ohio State University Comprehensive Cancer Center, Columbus, Ohio

**Keywords:** Central lung, Ultracentral lung, MR-guided radiotherapy, Stereotactic ablative, Radiotherapy

## Abstract

**Introduction:**

The recent results from the Nordic-HILUS study indicate stereotactic body radiation therapy (SBRT) is associated with high-grade toxicity for ultracentral (UC) tumors. We hypothesized that magnetic resonance-guided SBRT (MRgSBRT) or hypofractionated radiation therapy (MRgHRT) enables the safe delivery of high-dose radiation to central and UC lung lesions.

**Methods:**

Patients with UC or central lesions were treated with MRgSBRT/MRgHRT with real-time gating or adaptation. Central lesions were defined as per the Radiation Therapy Oncology Group and UC as per the HILUS study definitions: (1) group A or tumors less than 1 cm from the trachea and/or mainstem bronchi; or (2) group B or tumors less than 1 cm from the lobar bronchi. The Kaplan-Meier estimate and log-rank test were used to estimate survival. Associations between toxicities and other patient factors were tested using the Mann-Whitney *U* test and Fisher’s exact test.

**Results:**

A total of 47 patients were included with a median follow-up of 22.9 months (95% confidence interval: 16.4–29.4). Most (53%) had metastatic disease. All patients had central lesions and 55.3% (n = 26) had UC group A. The median distance from the proximal bronchial tree was 6.0 mm (range: 0.0–19.0 mm). The median biologically equivalent dose (α/β = 10) was 105 Gy (range: 75–151.2). The most common radiation schedule was 60 Gy in eight fractions (40.4%). Most (55%) had previous systemic therapy, 32% had immunotherapy and 23.4% had previous thoracic radiation therapy. There were 16 patients who underwent daily adaptation. The 1-year overall survival was 82% (median = not reached), local control 87% (median = not reached), and progression-free survival 54% (median = 15.1 mo, 95% confidence interval: 5.1–25.1). Acute toxicity included grade 1 (26%) and grade 2 (21%) with only two patients experiencing grade 3 (4.3%) in the long term. No grade 4 or 5 toxicities were seen.

**Conclusions:**

Previous studies noted high rates of toxicity after SBRT to central and UC lung lesions, with reports of grade 5 toxicities. In our cohort, the use of MRgSBRT/MRgHRT with high biologically effective doses was well tolerated, with two grade 3 toxicities and no grade 4/5.

## Introduction

Stereotactic body radiation therapy (SBRT) has been used for more than 20 years to treat early-stage nonoperable NSCLC.^1^, ^2^, ^3^ SBRT allowed increased doses per fraction and reduced treatment time, leading to widespread adoption. Since its implementation, multiple studies have reported excellent local control (LC) (≥90% at 3 y) and minimal toxicity rates (≤4% grade 3 or higher).^3^, ^4^, ^5^ The use of SBRT was not limited to the treatment of primary lung cancer as multiple studies have investigated its role in the treatment of metastatic disease to the lung and reported very high rates of LC.^6^^,^^7^

However, more grades 3 to 5 toxicities were seen in centrally located tumors as compared with more peripherally located tumors^8^, ^9^, ^10^ as defined as within 2 cm of the proximal bronchial tree (PBT) by the Radiation Therapy Oncology Group (RTOG).^10^ Subsequent studies found that fractionation mitigates toxicity in patients with central lesions as reflected in the American Society for Radiation Oncology’s SBRT Consensus.^3^, ^4^, ^5^^,^^8^

More recently, a subgroup of “ultracentral” lesions has been identified, which seem to carry an even higher risk of toxicity when treated with SBRT.^11^^,^^12^ Specifically, the Nordic-HILUS trial reports high rates of toxicity, with approximately 30% of patients experiencing grade 3 to 5 toxicities and 15% with treatment-related death.^12^ A systematic review by Chen et al.^13^ revealed that for 250 patients with ultracentral (UC) lung lesions, the risk of mortality is 5% (range: 0%–22%), with hemorrhage being the most common cause. These unacceptably high toxicity rates necessitate the use of a safer delivery technique if SBRT is to be used for the treatment of these lesions.

The advent of magnetic resonance (MR)-guided radiation therapy (MRgRT) has allowed for improved soft tissue visualization and real-time target tracking with beam gating, allowing for smaller margins, and daily on-table treatment adaptation to facilitate isotoxic dose escalation. We sought to investigate the role of MR-guided SBRT (MRgSBRT) and MR-guided hypofractionated radiation therapy (MRgHRT) in central and UC lung lesions. We hypothesized that, with the advantages of MRgSBRT and MRgHRT, we could achieve excellent LC with minimal toxicity.

## Materials and Methods

### Patient Eligibility

**Figure 1:**
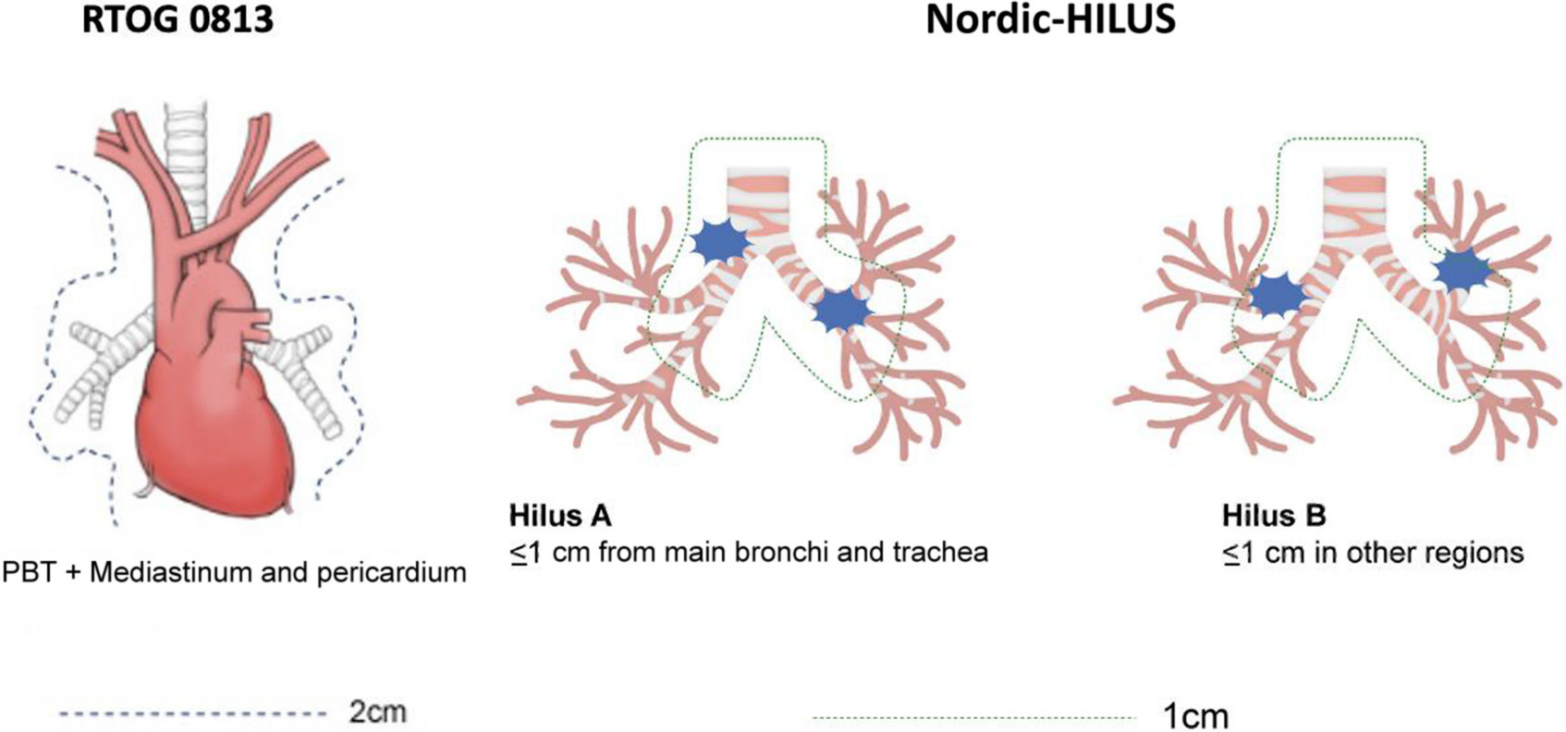
Left diagram depicting RTOG 0813 definition of UC lung lesions as within 2 cm from the PBT, or immediately adjacent to mediastinal and pericardial pleura. Right diagram depicting Nordic-HILUS definition of group A lesions within 1 cm of trachea and mainstem bronchi and group B lesions within 1 cm in other areas (e.g., lobar bronchus). PBT, proximal bronchial tree; RTOG, Radiation Therapy Oncology Group; UC, ultracentral.

After obtaining institutional review board approval, 47 patients were identified who were treated with MRgSBRT or MRgHRT for central or UC lung tumors between March 2019 and August 2021. Central tumors were defined as per RTOG 0813.^10^ UC tumors were defined as per Nordic-HILUS and subclassified into groups A and B^12^ (Fig. 1). Patients with primary lung cancers and metastatic lesions to the lung were included.

### MRgRT Planning and Treatment

Patients underwent magnetic resonance imaging (MRI) simulation in the 0.35T MRIdian System (ViewRay Inc., Mountain View, CA) along with computed tomography (CT) simulation for tissue density information. MRI and CT scans were each done in the supine position with a deep inspiratory breath hold scan. During MRI simulation, a balanced steady-state progression (TrueFISP [ViewRay, Inc.]) sequence was used to create images weighted by T2/T1 ratio. No immobilization devices were used.

The gross tumor volume (GTV) was identified as the tracking structure. A 3-mm isotropic expansion was used to create a nominal planning target volume (PTV) and tracking boundary structure for real-time gating during treatment delivery. The gating boundary structure was completely encompassed by the PTV to ensure appropriate dosimetric coverage. A 25- to 30-second cine sequence was obtained while the patient was performing a cycle of breath hold and free breathing maneuvers to ensure appropriate tracking and duty cycle for treatment delivery. A percentage excursion threshold of the tracking structure outside of the boundary structure was typically set at less than 5% to trigger beam-on, as previously described.^14^ No internal target volume was generated, allowing for the treatment of small volumes and, thereby facilitating reduced dose to surrounding critical structures.

Plans were prescribed to cover 95% of the PTV with at least 95% of the prescription dose, subject to strict organ-at-risk (OAR) constraints. Internal hot spots were limited to 125% of the prescription dose given the proximity to critical structures. Decision-making for daily adaptation was on the basis of the potential to improve GTV coverage, the need to cure OAR constraint violations, or both, on the basis of daily setup and anatomical shifts. Changes to improve GTV coverage were regulated to avoid violation of critical OAR structures. All plans used step-and-shoot intensity-modulated radiation therapy (RT) with the dose calculated on a 2-mm isotropic dose grid through the MRIdian Monte Carlo algorithm. The dose engine treats voxels with different CT numbers as water of variable density to account for heterogeneity.

### Toxicity Analysis

Toxicities were assessed using Common Terminology Criteria for Adverse Events version 5.0. Acute toxicities were defined from the start of treatment to shorter than 60 days after treatment. Late toxicities were defined as 60 days or longer from the end of treatment. Data were collected retrospectively for possible treatment-related toxicities.

### Statistical Analysis

Survival statistics were estimated using Kaplan-Meier analysis and variables were compared using the log-rank test. Multivariable Cox regression was used when appropriate. Associations between the incidence of toxicities and other patient factors were tested using the Mann-Whitney *U* test for continuous variables and Fisher’s exact test for categorical variables. LC was defined as the absence of local failure (LF) as per Response Evaluation Criteria in Solid Tumors confirmed by CT, positron emission tomography, or biopsy marginal failure or involved lobe failure, as per RTOG 0813.^10^ Overall survival (OS) was defined from the completion of SBRT to the time of death or last known follow-up. Progression-free survival (PFS) was defined as time to local or distant recurrence from completion of treatment.

## Results

### Patient and Treatment Characteristics

Patient and treatment characteristics are summarized in Table 1. A total of 47 patients were included in this study. The median age at treatment was 67.3 years (range: 32.3–79.2) and 51% were women. Most had good performance status (93.6% Eastern Cooperative Oncology Group 0–1). In this cohort, 22 of 47 patients had primary NSCLC histological diagnosis including adenocarcinoma, squamous cell, carcinoid, neuroendocrine carcinoma, and presumed NSCLC. The most common primary lung tumors were squamous cell (n = 8), and adenocarcinoma (n = 7). The remaining 53% had metastatic disease to the lung from a variety of primary histologies including thymic carcinoid, mesothelioma, melanoma, colorectal adenocarcinoma, pancreatic adenocarcinoma, salivary duct carcinoma, sarcoma, nonmelanoma cutaneous carcinoma, and renal cell carcinoma (Table 1), with the most common metastatic histologies being colorectal (n = 7) and melanoma (n = 6). All patients in this study had central lung lesions as defined by RTOG 0813.^10^ On further subclassification, 38 of 47 patients had UC lesions per HILUS, with 55.3% of patients having HILUS group A lesions. Patients with central lesions (<2 cm from PBT) but not meeting the criteria for UC were labeled as non-UC. The median distance to PBT in this cohort was 6 mm (range: 0.0–19.0). The median biologically effective dose (BED)_(a/b = 10)_ delivered was 105 Gy (range: 75.0–151.2). Before undergoing MRgRT, 26 patients (55.3%) received systemic therapy, of which 15 (31.9%) had immunotherapy (IO): ipilimumab in combination with nivolumab (n = 5) or with pembrolizumab (n = 3), pembrolizumab alone (n = 3), nivolumab alone (n = 2), atezolizumab (n = 1) or pantimumab (n = 1). Of note, 11 (23.4%) patients had previous thoracic RT. Four of 47 patients had concurrent IO; no patients received concurrent cytotoxic systemic therapy. A total of 34% of patients underwent daily treatment adaptation.

**Table 1:** Patient and Treatment Characteristics (N = 47)

		No. of Patients	%
Age, y			
Median	67.3		
Range	32.3–79.2		
IQR	59.7–74.1		
Sex			
Male		23	48.9
Female		24	51.1
ECOG			
0		15	31.9
1		29	61.7
2		3	6.4
Histology			
NSCLC Histologies		22	46.8
Non-NSCLC Histologies		25	53.2
Location			
RTOG		47	100.0
HILUS A		26	55.3
HILUS B		12	25.5
Distance to PBT (mm)			
Median	6.0		
Range	0.0–19.0		
IQR	1.0–9.0		
Target volumes (cm^3^)			
GTV volume			
Median	9.14		
Range	1.9–136.6		
IQR	5.1–19.6		
PTV volume			
Median	21.41		
Range	3.1–183.0		
IQR	13.8–41.6		
BED (α/β=10; Gy)			
Median	105.0		
Range	75.0–151.2		
IQR	100.0–105.0		
Treatment days			
Median	9		
Range	4–29		
IQR	8–12		
Prior systemic therapy			
Yes		26	55.3
No		21	44.7
Prior immunotherapy			
Yes		15	31.9
No		32	68.1
Prior thoracic RT			
Yes		11	23.4
No		36	76.6
Concurrent IO therapy			
Yes		4	8.5
No		43	91.5
Daily adaptation			
Yes		16	34.0
No		31	66.0
Adapted fractions			
Median Fx adapted	7		
Range Fx adapted	1–8		

*Note:* Non-NSCLC histologies includes colorectal (n = 7), melanoma (n = 6), renal cell (n = 3), nonmelanoma cutaneous (n = 2), salivary (n = 2), breast (n = 1), thymic (n = 1), mesothelioma (n = 1), pancreas (n = 1), sarcoma (n = 1). NSCLC histologies include squamous cell (n = 8), adenocarcinoma (n = 7), presumed NSCLC (n = 4), neuroendocrine (n = 2) and carcinoid (n = 1).

BED, biologically effective dose; Fx, fractions; ECOG, Eastern Oncology Cooperative Group; GTV, gross tumor volume; IQR, interquartile range; IO, immunotherapy; PBT, proximal bronchial tree; PTV, planning treatment volume; RT, radiotherapy; RTOG, Radiation Therapy Oncology Group.

Table 2 summarizes the different radiation dose and fractionation schedules used according to the location of the lesion. In this study, fractionation regimens varied from 3 to 15 fractions, with the most typically used being 60 Gy in eight fractions. Patients with UC lesions (HILUS A and B) were most frequently treated in eight fractions (50% and 42%, respectively). Approximately 30% of HILUS A patients received longer hypofractionated regimens (10– 5 fractions) compared with HILUS B (8.3%) or non-UC (11.1%). In addition, whereas five fraction regimens were used in HILUS A (19.2%) it was more typically used for the treatment of HILUS B (41.6%) and non-UC patients (44.4%).

**Table 2:** Radiation Regimen Utilized According Location of Lesion

	No. of Patients	%
54 Gy in 3 Fx		
HILUS A	0	0.0
HILUS B	1	8.3
Non-UC	3	33.3
50 Gy in 5 Fx		
HILUS A	2	7.7
HILUS B	4	33.3
Non-UC	1	11.1
60 Gy in 5 Fx		
HILUS A	3	11.5
HILUS B	1	8.3
Non-UC	3	33.3
60 Gy in 8 Fx		
HILUS A	13	50.0
HILUS B	5	41.7
Non-UC	1	11.1
50 Gy in 10 Fx		
HILUS A	1	3.8
HILUS B	1	8.3
Non-UC	0	0.0
60 Gy in 15 Fx		
HILUS A	7	26.9
HILUS B	0	0.0
Non-UC	1	11.1

Fx, fraction; Non-UC, nonultracentral.

### Outcomes

**Figure 2:**
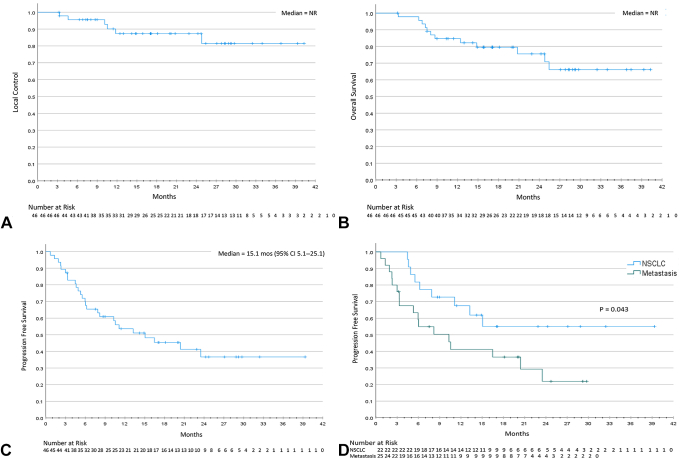
Survival outcomes for the entire cohort and by histologic classification. Kaplan-Meier curves for (*A*) local control, (*B*) overall survival, (*C*) progression-free survival, and (*D*) progression survival stratified by histology. CI, confidence interval; NR, not reached.

**Figure 3:**
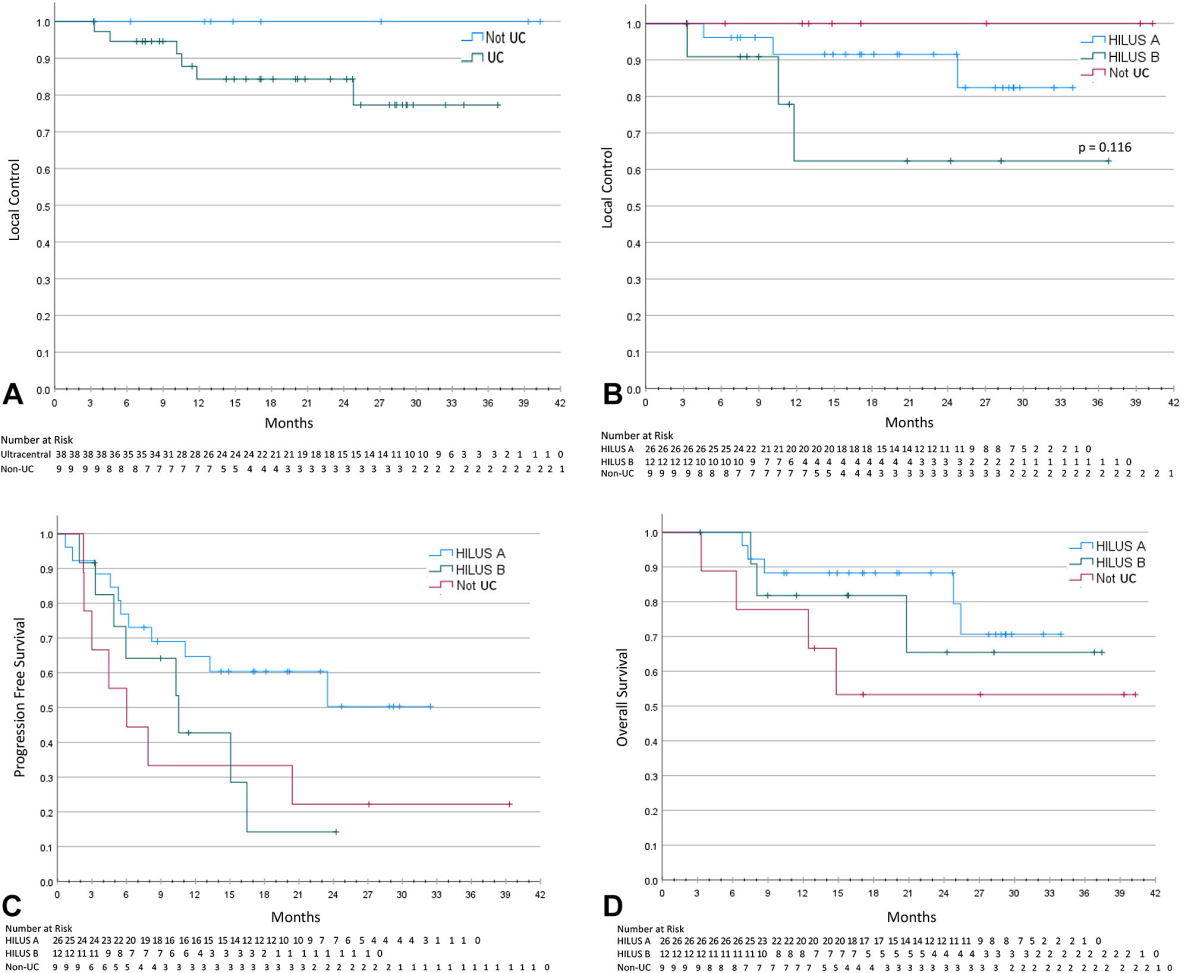
Survival outcomes stratified by location. Kaplan-Meier curves for local control (*A*) stratified by UC versus non-UC, (*B*) HILUS group A, B or non-UC, (*C*) progression-free survival, and (*D*) overall survival stratified by HILUS group A, group B, or non-UC. UC, ultracentral.

The median follow-up was 22.9 months (95% confidence interval [CI]: 16.4–29.4). Across all patients, the 1-year LC was 87% (median = not reached), and OS was 82% (median = not reached) (Fig. 2*A* and *B*). The 1-year PFS was 54% (median = 15.1 mo, 95% CI: 5.1–25.1) (Fig. 2*C*). Metastatic disease histology was associated with worse PFS (*p* = 0.043) as illustrated in Figure 2*D*. Supplementary Table 1 summarizes the variables tested for their association with PFS using the log-rank test. Variables with a *p* value threshold of less than 0.05 were selected for Cox regression analysis, of which, histology and late toxicity initially seemed as statistically significant. However, on multivariable Cox regression, statistical significance was not achieved between PFS and metastatic disease histology (hazard ratio = 1.7, 95% CI: 0.76–3.81, *p* = 0.201) or late toxicity (*p* = 0.973) (Supplementary Table 1). Next, we sought to assess factors associated with LC. We stratified patients by UC versus non-UC; however, there were no LF events in the non-UC group (all cases censored at the time of the last follow-up) and only six in the UC cohort; therefore, no valid statistics can be computed (Fig. 3*A*). On further review of these patients, all failures occurred within the primary treated tumor and were diagnosed radiographically in five and with biopsy in one patient. Outcomes were then stratified by HILUS category A, B, and non-UC, as depicted in Figure 3*B* to *D*. Overall, no statistically significant correlations were identified in terms of LC (*p* = 0.116), OS (*p* = 0.318) or PFS (*p* = 0.105). We also explored the following factors on survival outcomes: sex, Eastern Cooperative Oncology Group status, previous treatments (including local, systemic, or IO), daily adaptation, BED, and acute/late toxicity. However, no statistically significant differences were detected.

### Toxicity

Toxicities are summarized in Table 3. Acute toxicity was defined as adverse events possibly related to treatment occurring shorter than 60 days from completion, whereas late toxicity was defined as events occurring 60 days or longer. A total of 42 patients experienced acute toxicities, which included grade 1 (n = 12, 25.5%) and grade 2 (n = 10, 21.3%) toxicities. The most common grade 1 toxicity observed included fatigue and cough. In the patients that developed grade 2 adverse events, the most typically reported were esophagitis and dyspnea. Of note, two patients developed grade 2 pneumonitis, which required outpatient treatment with oral steroids with improvement. In the acute setting, there were no grade 3 or higher adverse events. In the late setting, only two patients continued to experience grade 1 toxicity, namely fatigue and dyspnea. We observed two events (4.3%) of grade 3 toxicity, but no grade 4 or 5 events. In the patients that developed grade 3 toxicities, one developed esophagitis requiring hospital admission for pain control; however, a feeding tube was not required and his symptoms resolved completely about 7 months after RT. A second patient developed radiation pneumonitis 3 months after completion of treatment and required hospital admission for steroids and supplemental oxygen but did not require intubation. Of note, this patient had received SBRT to the same lesion 18 months prior with composite V20 of 6.2% (goal composite V20 <10%).

**Table 3:** Toxicity Profile

Acute toxicity	No. of Patients	%	Late toxicity	No. of Patients	%
Grade 1	12	25.5	Grade 1	2	4.3
Grade 2	10	21.3	Grade 2	1	2.1
Grade 3	0	0.0	Grade 3	2	4.3
Grade 4	0	0.0	Grade 4	0	0.0
Grade 5	0	0.0	Grade 5	0	0.0

Adverse event	Grade 1	Grade 2	Grade 3	Adverse event	Grade 1	Grade 2	Grade 3
	n	n	n		n	n	n
Fatigue	5	2	-	Fatigue	1	-	-
Dermatitis	3	-	-	Dermatitis	-	-	-
Anorexia	-	1	-	Anorexia	-	-	-
Noncardiac chest pain	1	-	-	Noncardiac chest pain	-	-	-
Dyspnea	3	3	-	Dyspnea	1	1	-
Cough	5	-	-	Cough	-	-	-
Pneumonitis	-	2	0	Pneumonitis	-	-	1
Esophagitis	-	4	0	Esophagitis	-	-	1

*Note:* Acute toxicity defined as occuring as <60 days from completion of treatment; Late toxicity defined as occurring ≥60 days after completion of treatment.

On initial analysis, we noticed an association between G2 toxicity and adaptation (*p* = 0.029) using Fischer’s exact test but was no longer significant after Bonferroni correction for multiple testing. We observed that grade 2 toxicity was associated with higher GTV volume (7.66 cm^3^ versus 15.66 cm^3^, *p* = 0.015) but not with PTV volume (*p* = 0.167) (Supplementary Figure 1). Otherwise, no associations were identified between grades 1 to 3 toxicity and sex, histology, HILUS groups, previous/concurrent therapy, or treatment adaptation.

## Discussion

The use of SBRT in the setting of centrally located lesions remains an area of controversy with conflicting evidence regarding its toxicity profile, with some studies reporting grade 3 to 5 toxicities as high as 30%.^12^^,^^13^ In this work, we report the safe delivery of MRgSBRT and MRgHFRT to central and UC lung lesions with minimal toxicity in contrast to other studies. Technological developments such as MRgRT provides a new avenue to approach the management of central and UC lung lesions by allowing the potential for ablative dose delivery within a narrow therapeutic window. Because of its improved soft tissue visualization compared with CT images, prospective studies have been designed to evaluate the safety of MRgRT in the ablative treatment of thoracic and abdominal malignancies.^15^^,^^16^ In the SMART study published by Washington University, five patients with both nonoperative early-stage NSCLC and oligometastatic disease with UC lesions were treated using MRgSBRT to 50 Gy in five fractions with excellent LC and no acute grade 3 or higher toxicities.^16^

In our cohort, the median follow-up time was 22.9 months (95% CI: 16.4–29.4), and the median LC and OS were not reached (Fig. 2*A* and *B*). The median PFS was 15.1 months (95% CI: 5.1–25.1) (Fig. 2*C*). We observed a trend that the previous use of IO was associated with inferior LC; however, it was not statistically significant (*p* = 0.06). We did not find a difference in terms of LC, OS, or PFS when patients were stratified according to the HILUS category (Fig. 3*B* and *C*). In our cohort, only six patients experienced LF with all events occurring in patients with UC lesions. As represented in Figure 3*B*, patients with HILUS B lesions seemed to have inferior LC; however, it was not statistically significant (*p* = 0.116). On further analysis of these patients, we were not able to identify high-risk features that would account for this in terms of BED, GTV/PTV volume, pretreatment diameter, or proportion of metastatic versus primary lung when compared with HILUS A alone. When the non-UC patients were added to the analysis, there was a statistically significant difference in BED (*p* = 0.042); however, this was primarily driven by the differences in HILUS A and non-UC on pairwise analysis.

The association with worse PFS and inferior LC in patients with metastatic histology, albeit not reaching statistical significance (Supplementary Table 1), highlights the importance of patient selection when pursuing aggressive ablative therapies.

The most common acute toxicities reported by patients in this study include grade 1 and grade 2, with fatigue being the most common. In addition, whereas a little over 90% (42 of 46) of patients experienced some degree of treatment-related toxicity in the acute setting, most experienced near-complete relief at 2 months after treatment, with only 9% (4 of 46) having late toxicity (Table 3). Our results are consistent with the published data reporting development of toxicity from SBRT is generally mild (grade 1–2) and resolves within 3 to 4 months, particularly in the setting of peripheral lung lesions.^8^ However, in the setting of UC lesions, there is extensive literature documenting the increased rates of severe toxicity, particularly within the first 2 years.

In the dose escalation trial for SBRT in central lung lesions, severe toxicity (grade ≥3) including bronchopulmonary hemorrhages and respiratory failures occurred with delayed onset. RTOG 0813 exemplifies this well; although they had a predetermined 1-year toxicity end point, serious adverse events persisted for longer than 1 year, including the formation of an esophageal ulcer, which eroded into a major vessel and bronchopulmonary hemorrhages.^10^ In a recent retrospective study of SBRT in UC, central, and paramediastinal lesions, the authors reported a 2-year risk of grade 2 or higher toxicity of 57.5%, 14.2%, and 7.1% respectively.^17^ This group also reported delayed grade 5 events.^17^ The Nordic-HILUS study reported a 34% rate of development of grade 3 to 5 toxicities, and most significantly, a 15% rate of treatment-related death, which was more common in HILUS A patients, and peaked between 1 and 2 years after treatment.^12^ Whereas these studies serve as a warning light regarding the management of central and UC lung lesions, these studies all used CT-based planning.

At a median follow-up of 22.9 months, we observed a 4.3% rate of grade 3 toxicity with no grade 4 or 5 adverse events (Table 3). The patients in our cohort are within the time frame in which HILUS and other studies have reported a peaked incidence of severe toxicity, and this is further highlighted by the association between grade 3 adverse events and late toxicity (*p* = 0.009). In addition, more than half of the patients met the HILUS A criteria in which the authors reported the highest rate of fatal bronchopulmonary hemorrhage, although using MRgRT exhibited no increased toxicity for such patients. In contrast to HILUS, we limit our internal maximum point dose to 125% of the prescription dose, rather than 150% (prescribed to the 67% isodose line in Nordic-HILUS). MR guidance also allowed us to avoid the creation of an internal target volume, leading to treatment volume reductions. In addition, select cases including a large proportion of HILUS A patients at our institution were treated with longer hypofractionated regimens spanning 10 or 15 fractions, instead of eight. All three of these factors likely influenced the differences seen in toxicities.

Some limitations of the current study include the inherent bias of retrospective study, particularly around the decision of the treating physician to pursue an adaptive course versus a nonadaptative. Because OAR constraints were set a priori, it is possible that this was less subjective to bias, whereas the GTV coverage decision was more likely to be subjective. In addition, the presence of mixed histologies, metastatic versus primary disease, and the variation in dose and fractionation regimens complicates the analysis of efficacy and safety. Finally, another limitation is the single institution and small sample size. Nevertheless, this study is one of the largest to report on the use of MRgRT for patients with high-risk lung lesions. Overall, these results highlight that MRgRT allowed good targeting to minimize the risk of higher than grade 3 adverse events and permitted treatment of central and UC lesions with manageable toxicities. Given these promising results, we are co-leading a trial of adaptive MRgRT for central and UC lung tumors to test efficacy in a prospective manner (Lung STAAR; NCT04917224).

In conclusion, our study found that MRgSBRT or MRgHRT can be safely used for the treatment of central and UC lesions in patients with early-stage NSCLC or oligometastatic disease in the lungs. This technique may allow the treatment of central and UC lesions to high ablative doses while minimizing the risk of severe grade 3 or higher toxicity and is currently being evaluated in a prospective study to validate the safety and effectiveness.

## CRediT Authorship Contribution Statement

**Maria Sandoval:** Conceptualization, Data collection, Data analysis, Writing – original draft preparation.

**Austin Sim:** Conceptualization, Data collection, Data analysis, Writing – original draft preparation.

**John Bryant:** Data collection, Data curation, Writing – review/ editing.

**Menal Bhandari:** Data collection, Data curation.

**Thomas Dilling:** Conceptualization, Methodology, Writing – review/editing.

**Bradford Perez:** Methodology, Writing – review/editing.

**Evan Wuthrick:** Methodology, Writing – review/editing.

**Gage Redler:** Supervision, Writing – review/editing.

**Jacqueline Andreozzi:** Supervision, Writing – review/editing.

**Louis Nardella:** Supervision, Writing – review/editing.

**Vladimir Feygelman:** Supervision, Writing – review/editing.

**Kujtim Latifi:** Supervision, Writing – review/editing.

**Stephen Rosenberg:** Conceptualization, Administration, Supervision, Writing – review/editing.
