# Supplementary material for: Magnetic Resonance-Guided Stereotactic Body Radiation Therapy/Hypofractionated Radiation therapy for Metastatic and Primary Central and Ultracentral Lung Lesions
Source: JTO Clin Res Rep. 2023 Feb 25;4(5):100488. doi: 10.1016/j.jtocrr.2023.100488 (PMC10163640; doi:10.1016/j.jtocrr.2023.100488)
Supplement: Supplementary Table [file mmc1.docx]

**Supplemental Data**

**Figure 1-S.**

*Figure 1-S:* Toxicity associations. Mann Whitney U test for Grade 1 toxicity (A) distance from PBT, (B) treatment duration, (C) GTV volume and (D) PTV volume. Grade 2 toxicity (E) distance from PBT, (F) treatment duration, (G) GTV volume and (H) PTV volume.
